# TREM2 Regulates High Glucose-Induced Microglial Inflammation via the NLRP3 Signaling Pathway

**DOI:** 10.3390/brainsci11070896

**Published:** 2021-07-07

**Authors:** Yuan Li, Weihong Long, Menghan Gao, Fangtai Jiao, Zecai Chen, Mingyuan Liu, Lu Yu

**Affiliations:** 1 Key Laboratory of Zoonosis Research, Ministry of Education, Institute of Zoonosis, College of Veterinary Medicine of Jilin University, Changchun 130000, China; yuanli18@mails.jlu.edu.cn (Y.L.); longwh9918@mails.jlu.edu.cn (W.L.); gaomh9919@mails.jlu.edu.cn (M.G.); jiaoft9916@mails.jlu.edu.cn (F.J.); chenzc9916@mails.jlu.edu.cn (Z.C.); liumy36@163.com (M.L.); 2 Jiangsu Co-Innovation Center for Prevention and Control of Important Animal Infectious Diseases and Zoonosis, Yangzhou 225009, China

**Keywords:** diabetes, microglia, TREM2, NLRP3

## Abstract

**Background:** TREM2 expressed on microglia plays an important role in modulating inflammation in neurodegenerative diseases. It remains unknown whether TREM2 modulates hyperglycemia-induced microglial inflammation. **Methods:** We investigated the molecular function of TREM2 in high glucose-induced microglial inflammation using western blotting, qPCR, ELISA, pulldown, and co-IP methods. **Results:** Our data showed that in high glucose-induced BV2 cells, TREM2 was increased, and the proinflammatory cytokine IL-1β was increased. TREM2 knockout (KO) attenuated the proinflammatory cytokine IL-1β; conversely, TREM2 overexpression (OE) exacerbated IL-1β expression. Furthermore, we found that high glucose promoted the interaction of TREM2 with NLRP3. TREM2 KO abolished the interaction of TREM2 with NLRP3, while TREM2 OE enhanced the interaction. Moreover, TREM2 KO reduced high glucose-induced NLRP3 inflammasome activation, and TREM2 OE augmented high glucose-induced NLRP3 inflammasome activation, indicating that high glucose enhances the expression of TREM2, which activates the NLRP3 inflammasome. To further clarify whether the NLRP3 signaling pathway mediates the TREM2-regulated inflammatory response, we blocked the NLRP3 inflammasome by knocking out NLRP3 and treating cells with a caspase1 inhibitor, which decreased the levels of the IL-1β proinflammatory cytokine but did not affect the high glucose-induced expression of TREM2. **Conclusions:** TREM2 modulates high glucose-induced microglial inflammation via the NLRP3 signaling pathway.

## 1. Introduction

Diabetes has become a major chronic epidemic worldwide and is associated with changes in lifestyle, obesity, lack of exercise, and increased longevity [1]. However, glucose and lipid metabolism disorders caused by defects in insulin secretion and reduced responses to insulin-stimulated glycometabolism are the main pathological features of diabetes. Recent studies have found that metabolic disorder-induced glucotoxicity, oxidative stress, lipotoxicity, and endoplasmic reticulum stress can lead to systemic chronic inflammation that exacerbates the course of diabetes and diabetes-associated complications [2,3]. Increased activation of proinflammatory factors, such as IL-1β, has been reported to cause defective insulin secretion and insulin resistance [4]. In the central nervous system, microglia play an important role in defending the brain against invading microorganisms and metabolic stress. Microglia are divided into the classical M1 phenotype and the alternative M2 phenotype [5]. The M1 phenotype shows an amoeboid morphology and high phagocytic activity that can remove harmful factors and apoptotic neuronal debris by releasing a range of proinflammatory cytokines and chemicals once stimulated by invading pathogens or disturbing the CNS environment. In contrast, the M2 phenotype shows a ramified morphology by secreting neurotrophic and antinflammatory factors to maintain homeostasis of the CNS environment [6]. Suppression of the microglial M1 phenotype and promotion of the transformation to the M2 phenotype have been reported to ameliorate chronic neurological disorders, such as AD [7], Parkinson’s disease [5], stroke [8], LPS-induced neuroinflammation [9], and exposure to electromagnetic field-induced neuroinflammation [10]. Studies have shown that microglia are in a state of overactivation in diabetic cognitive decline attributed to disordered glucose metabolism [11]. Activated microglia are also associated with neurodegeneration, including hippocampal injury and cerebral atrophy, in animal models of diabetes and diabetes patients [12,13]. Furthermore, researchers have found that hyperglycemia increases microglial vulnerability to lipopolysaccharide (LPS)-induced inflammation [14]. In cultured microglia, high glucose stimulates microglial activation via the ROS and NF-κB pathways [15]. Some research has demonstrated that inhibition of microglial activation attenuates diabetes-induced inflammatory cytokine production and reduces apoptosis [16]. Thus, elucidating the mechanism and suppression of microglia activation in high-glucose stress is important for hyperglycemia-induced neuroinflammatory diseases.

TREM2 is a type of immunoglobulin receptor highly expressed on microglial cells and plays a critical role in the negative regulation of autoimmune and inflammatory processes [17]. TREM2 interacts with its adaptor protein, DAP12, to transduce signals. Rare variants of TREM2 are associated with the occurrence of Alzheimer’s disease and Nasu-Hakola disease (NHD), and other neurological diseases, including PLOSL, frontotemporal dementia, and Parkinson’s disease [18]. Moreover, recent studies have shown that TREM2 expressed on microglia is responsible for synaptic elimination and normal brain connectivity [19], maintaining the balance of metabolism and innate immunity [20,21]. Therefore, we investigated whether TREM2 is involved in the pathogenesis of diabetes-associated cognitive decline to provide a new target for the treatment of diabetes-associated neuroinflammation.

The NLRP3 inflammasome has been implicated in the development of diabetes and neurodegenerative diseases [22]. Our previous study found that high glucose stimulates microglial NLRP3 inflammasome activation via the ROS/JNK MAPK/NF-κB pathway in vivo and in vitro and that the suppression of NLRP3 inflammasomes ameliorates high glucose-induced proinflammatory cytokine release (manuscript accepted for publication). We hypothesized that TREM2 regulates high glucose-induced microglial inflammation via the NLRP3 inflammasome pathway. We aimed to first determine the relationship between TREM2 and the proinflammatory cytokine, IL-1β, to understand the molecular pathway of TREM2 in regulating high glucose-induced neuroinflammation. We then investigated whether TREM2 functions in the activation of the NLRP3 inflammasome. Finally, we evaluated the inflammatory response after blocking the NLRP3 signaling pathway. Overall, we aimed to elucidate the modulation pathway of TREM2 in high glucose-induced neuroinflammation.

## 2. Materials and Methods

### 2.1. Cell Culture

The BV2 cell line was maintained in our lab. Cells were cultured in low-glucose DMEM (5.5 mmol/L) (HyClone, Logan, USA, Catalog No. SH30021.01) containing 10% FBS (Biological Industries, Israel, Catalog No. 04-001-1ACS) and 1% penicillin-streptomycin (Gibco Life Technologies, Grand Island, USA, Catalog No. 15140122) at 37 °C in an atmosphere of 5% CO2. Cells (1 × 10^6^) were treated with different concentrations of D-glucose (Sigma-Aldrich, Saint Louis, USA, Catalog No. 154-17-6) for different time courses.

### 2.2. Western Blot Analysis

Western blotting was performed as previously described [23]. The following antibodies were used: anti-TREM2 (1:1000) (Abcam, Cambridge, England, Catalog No. ab125117), anti-NLRP3 (1:1000) (Cell Signaling Technology, Beverly, USA, Catalog No. 15101S), anti-cleaved caspase1 (1:1000) (Cell Signaling Technology, Beverly, USA, Catalog No. 89332S), anti-cleaved IL-1β (1:1000) (Cell Signaling Technology, Beverly, USA, Catalog no. 63124S), and anti-GAPDH (1:2000) (Bioworld, Minnesota, USA, Catalog No. AP0066).

### 2.3. qRT-PCR

Total RNA was extracted from cultured cells (10^6^) using a total RNA extraction kit (QIAGEN, Beijing, China, Catalog No. 90001), and reverse transcription was performed using an EasyScript^®^ Reverse Transcriptase kit (TransGen Biotech, Beijing, China, Catalog No. AE301-02). Quantitative real-time PCR (qRT-PCR) was performed using the Applied Biosystems 7900HT fast real-time PCR system and SYBR Green PCR master mix (Roche, Basel, Switzerland, Catalog No. 4913850001). The following primers were used: TREM2, 3′-CAGCACCTCCAGGAATCAAGA-5′ and 5′-AGGATCTGAAGTTGGTGCCC-3′; and IL-1β, 3′-TGCCACCTTTTGACAGTGATG-5′ and 5′-AAGGTCCACGGGAAAGACAC-3′. All reactions were performed in triplicate, and each experiment was repeated three times. The relative expression of each target gene was calculated using the 2-ΔΔCt method.

### 2.4. Generation of CRISPR/Cas9-Mediated Knockout (KO) Cell Line

CRISPR/Cas9-mediated gene editing was performed using a px459 vector (Addgene, Catalog No. 48139) targeting murine TREM2 or NLRP3 in BV2 cells, and the plasmid construction has been previously described [24]. The sgRNAs targeting the TREM2 sequence (5′-TCCCAAGCCCTCAACACCA-3′) and NLRP3 sequence (5′-CAAGCTGGCTCAGTATCTAG-3′) were synthesized by Comate Bioscience Company (Shanghai, China). According to the manufacturer’s instructions, plasmids were transfected with Attractene Transfection Reagent (QIAGEN, Beijing, China, Catalog No. 301004). Clonal lines were established by 96-well plate screening. The KO cell lines were verified by western blotting after the clones had formed. For the high glucose treatment experiment, KO cells were treated with high glucose at 35 mM for 12 h.

### 2.5. Generation of TREM2-Overexpressing (OE) Cell Line

The pCDNA3.1(+)-3×FLAG (Addgene, Catalog No. 105609) mammalian expression vector was used. The murine TREM2 gene was cloned from brain tissue cDNA using the following primers: forward primer, 5′-CCGGCTAGC ATGGGACCTCTCCACCAGTTTCTCC-3′; and reverse primer, 5′-CCGCTCGAG TCAGAATTCTCTCACGTACCTCCGG-3′. Both sequences were digested with the Nhe1 (NEB, Beijing, China, Catalog No. R3131S) and Xho1 (NEB, Beijing, China Catalog No. R0146S) restriction enzymes in a water bath for 3 h at 37 °C and then inserted into the pCDNA3.1(+)-3×FLAG vector to construct pCDNA3.1(+)-3×FLAG-TREM2 recombinant plasmids. After successful sequencing, the recombinant plasmids were transfected into BV2 cells using Attractene Transfection Reagent (QIAGEN, Beijing, China, Catalog No. 301004) according to the manufacturer’s instructions. The TREM2 expression level was determined by western blotting at 72 h after transfection. For the high glucose treatment experiment, OE cells were treated with high glucose at 35 mM for 12 h.

### 2.6. ELISA

Cells (10^6^) were incubated in 6-well plates and treated with or without 35 mM high glucose for 12 h. The cell supernatant was collected, and the IL-1β pro-inflammatory cytokine was detected using an ELISA kit according to the manufacturer’s instructions (Boster, Wuhan, China, Catalog No. EC0394).

### 2.7. GST Pull-Down Assay

PGEX4T-1 was purchased from the Public Protein/Plasmid Library (PPL, Catalog No. 27-4580-01) and used to construct the p-GEX4T1-λ-TREM2 plasmid. The PCR products targeting the *trem2* gene were amplified from the mouse cDNA library using the following primers: 5′-GGAATTCCTGCTGGCAAAGGAAAGGTG-3′ and 3′- CCTCGAGCTGGATTGACTCCTGGCTGG′. The PCR products and PGEX4T1λ were both digested by the EcoR1 (NEB, Beijing, China, Catalog No. R0101S) and Xho1 (NEB, Beijing, China, Catalog No. R0146S) restriction enzymes and used to construct the recombinant plasmid using T4 DNA ligase (NEB, Beijing, China, Catalog No. M0202S). The recombinant plasmids were transformed into an *E. coli* prokaryotic expression system to obtain purified GST-tagged TREM2 protein. Purified GST or GST-TREM2 proteins were incubated with glutathione-Sepharose beads (PureCube, Chengdu, China, catalog: 32103) at 4 °C for more than 2 h. The beads were washed three times with 1% Triton X-100 (Solarbio, Beijing, China, Catalog No. 9002-93-1) and added to BV2 cell lysate at 4 °C for 3 h. The beads were then extensively washed, and the bound proteins were eluted and separated by 10% SDS-PAGE for western blot analysis.

### 2.8. Coimmunoprecipitation

BV2 (10^8^) cells were collected and lysed with RIPA lysis buffer (1% NP-40 and 0.25% deoxycholate) (Beyotime, Beijing, China, Catalog No. P0013D) containing 1 mM phenylmethylsulfonyl fluoride (PMSF) (Beyotime, Beijing, China, Catalog No. ST505). Then, 30 μg of protein was added to 500 µl of RIPA lysis buffer, and 1 μg of the anti-TREM2 antibody (Abcam, Cambridge, England, Catalog No. ab125117) and 1 μg of normal rabbit IgG (Santa Cruz Biotechnology, Texas, USA, Catalog No. sc-2026) were added. Then, 30 μL of protein A/G beads (SMRRT, Changzhou, China, Catalog No. SA032005) was added to the protein-antibody mixture and incubated at 4 °C overnight. After incubation, the samples were centrifuged at 2500 rpm for 4 min at 4 °C and washed three times with RIPA lysis buffer. Next, the supernatant was removed, and 30 μL of 2× loading buffer was added and boiled for 10 min. The boiled samples were separated by 10% SDS-PAGE for western blot analysis using NLRP3 and TREM2 antibodies, and these immunoblot results were indicated as the IB (immunoblot) group. The 5% cell lysate was used as an input control (5% input), and it was blotted and analyzed with NLRP3, TREM2 and GAPDH antibodies.

### 2.9. Statistical Analysis

Data were analyzed by GraphPad Prism 8.0. Statistical significance was evaluated using independent sample one-way ANOVA or two-way ANOVA combined with post hoc tests for multiple comparisons. *p* < 0.05 was considered statistically significant.

## 3. Results

### 3.1. High Glucose Enhances the Expression of TREM2 and the IL-1β Proinflammatory Cytokine

**Figure 1:**
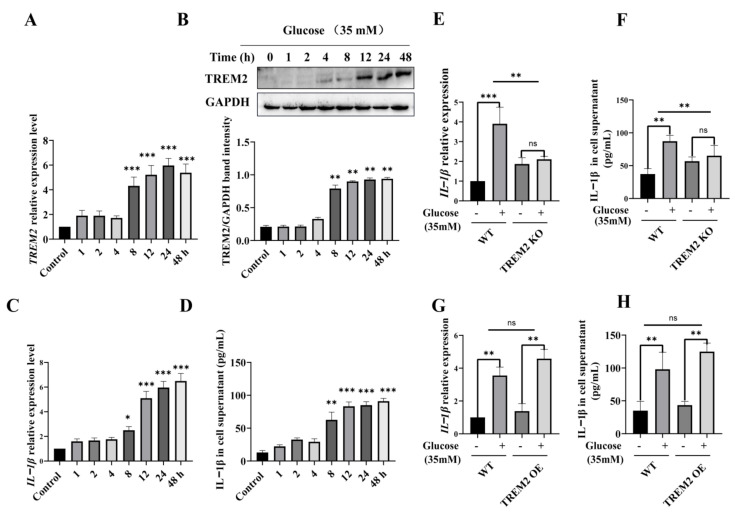
High glucose increases the transcription and expression of TREM2 and the IL-1β proinflammatory cytokine. (**A**) TREM2 transcription level after stimulation with high glucose (35 mM) for different time courses (1–48 h) in BV2 cells as detected by qPCR. The expression levels were normalized to GAPDH. (**B**) TREM2 expression levels were measured by western blotting after stimulation with high glucose (35 mM). The band intensity was normalized to GAPDH and analyzed using ImageJ software. (**C**) IL-1β transcription level after stimulation with high glucose (35 mM) in BV2 cells as detected by qPCR. The expression level was normalized to GAPDH. (**D**) ELISA detection of IL-1β in BV2 cells treated with high glucose for different time courses (1–48 h). In A-D, the data represent means ± SEM of 3 independent experiments. * *p* ≤ 0.05, ** *p* ≤ 0.01 and *** *p* ≤ 0.001 according to one-way ANOVA. (**E**) IL-1β transcription level after stimulation with or without high glucose (35 mM) for 12 h in TREM2 KO BV2 cells as detected by qPCR. The expression level was normalized to GAPDH. (**F**) ELISA detection of IL-1β in TREM2 KO BV2 cells treated with or without high glucose for 12 h. (**G**) IL-1β transcription level after stimulation with or without high glucose (35 mM) for 12 h in TREM2 OE BV2 cells as detected by qPCR. The expression level was normalized to GAPDH. (**H**) ELISA detection of IL-1β in TREM2 OE BV2 cells treated with or without high glucose for 12 h. In E-H, the data represent means ± SEM of 3 independent experiments. * *p* ≤ 0.05, ** *p* ≤ 0.01, and *** *p* ≤ 0.001 according to two-way ANOVA with Bonferroni’s post hoc test.

To determine whether TREM2 regulates high glucose-induced microglial inflammation, we used BV2 cells (immortalized primary microglia) treated with high glucose (35 mM) to mimic hyperglycemia in vitro. RT-PCR and western blotting or ELISA measured the transcription and expression levels of TREM2 and IL-1β upon high glucose treatment for different time courses (1 to 48 h). The results showed that the transcription and expression levels of TREM2 were both elevated after treatment for 8 h (Figure 1A,B), and the transcription and expression levels of IL-1β were increased after treatment for 8 h (Figure 1C,D), indicating that high glucose increases TREM2 expression and IL-1β proinflammatory cytokine levels. To further evaluate the proinflammatory cytokine release affected by TREM2 expression, we treated the TREM2-KO BV2 cell line constructed by the CRISPR/Cas9 method (Figure S1A–D) with high glucose for 12 h. The results showed that TREM2 KO ameliorated the transcription and expression of the IL-1β proinflammatory cytokine (Figure 1E,F). Moreover, we constructed a TREM2-OE BV2 cell line by transient transfection of pCDNA3.1-3*flag-TREM2 recombinant plasmids (Figure S2A–D), which was then stimulated with high glucose for 12 h. The results showed that TREM2 OE increased the transcription and expression of the IL-1β proinflammatory cytokine (Figure 1G–H). These results indicated that TREM2 protects against high glucose-induced neuroinflammation.

### 3.2. High Glucose Promotes the Interaction of TREM2 with NLRP3

**Figure 2:**
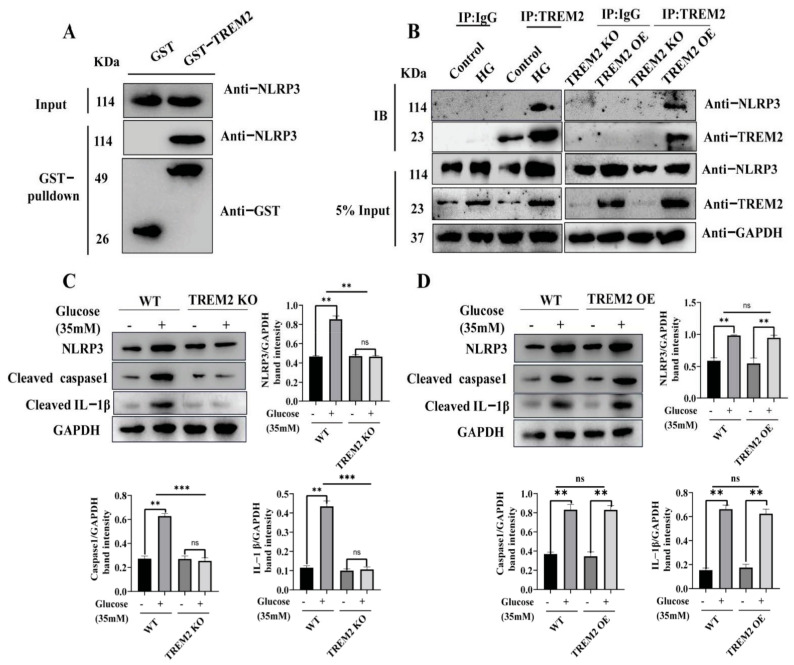
TREM2 enhances the activation of NLRP3 inflammasomes by interacting with NLRP3. (**A**) GST-TREM2 was expressed and purified from the *Escherichia coli* expression system, and cell lysates were precipitated with glutathione Sepharose beads and immunoblotted with an NLRP3 antibody. (**B**) Lysates from BV2 microglial cells were immunoprecipitated with a TREM2 antibody or rat IgG and immunoblotted with a mouse NLRP3 antibody. (**C**) Western blot analysis of NLRP3, caspase1, and IL-1β in WT control and TREM2-KO BV2 cells. GAPDH was used as an internal control for normalization. (**D**) Western blot analysis of NLRP3, caspase1, and IL-1β in WT control and TREM2-OE BV2 cells. GAPDH was used as an internal control for normalization. In C-D, the data represent means ± SEM of 3 independent experiments. ** *p* ≤ 0.01, and *** *p* ≤ 0.001 according to two-way ANOVA with Bonferroni’s post hoc test.

NLRP3 inflammasome activation has been reported to be responsible for the cleavage of pro-IL-1β into IL-1β. Thus, we investigated whether high glucose-induced TREM2 modulates the NLRP3 inflammasome. We used a GST pulldown assay to test whether TREM2 interacts with NLRP3, and the results demonstrated that TREM2 could interact with NLRP3 (Figure 2A). Furthermore, a co-IP method was used to test the interaction of TREM2 and NLRP3 in high glucose-treated WT, TREM2 KO, and TREM2 OE BV2 cells. The results showed that high glucose treatment enhanced the interaction of TREM2 and NLRP3. In addition, TREM2 KO abolished this interaction, and TREM2 OE enhanced this interaction (Figure 2B). Thus, these findings indicated that high glucose promotes the interaction of TREM2 and NLRP3. We evaluated the activation of the NLRP3 inflammasome in WT, TREM2 KO, and TREM OE BV2 cells treated with high glucose to understand the function of TREM2 in NLRP3 inflammasomes. The results showed that TREM2 KO alleviated the high glucose-induced expression of NLRP3, caspase1, and IL-1β(Figure 2C), and TREM2 OE enhanced the high glucose-induced expression of NLRP3, caspase1, and IL-1β (Figure 2D). These results demonstrated that TREM2 mediates the activation of the NLRP3 inflammasome.

### 3.3. TREM2-Regulated Microglial Inflammation Is Mediated by the NLRP3 Inflammasome Pathway

**Figure 3:**
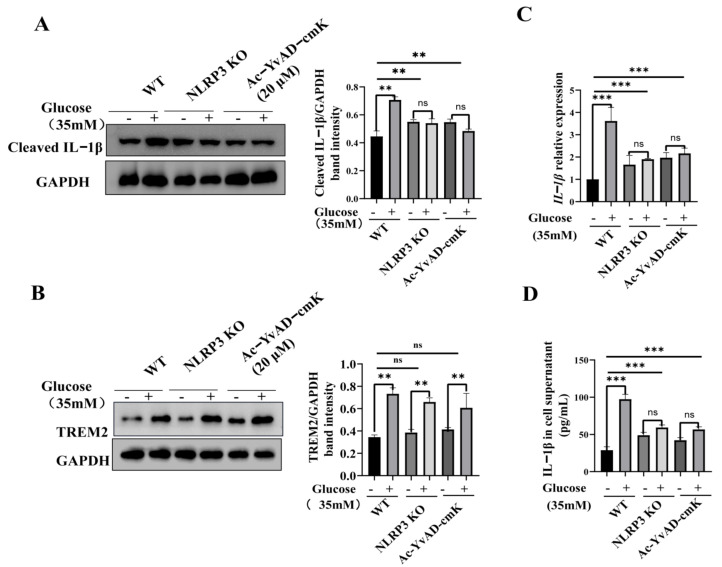
NLRP3 inflammasome suppression decreases the IL-1β proinflammatory cytokine. (**A**,**B**) Western blot analysis of IL-1β and TREM2 in WT control cells, NLRP3-KO cells, and WT cells treated with the caspase1 inhibitor, Ac-YvAD-cmK. GAPDH was used as an internal control for normalization. The values are expressed as the mean ± SEM. (**C**) qPCR detection of the IL-1β transcription level normalized to GAPDH. (**D**) ELISA detection of IL-1β in WT control and TREM2 OE BV2 cells. The data represent means ± SEM of 3 independent experiments. ** *p* ≤ 0.01, and *** *p* ≤ 0.001 according to two-way ANOVA with Bonferroni’s post hoc test.

To further examine whether the NLRP3 inflammasome specifically mediates TREM2-regulated microglial inflammation, we blocked NLRP3 activation by using CRISPR/Cas9-mediated NLRP3-KO BV2 cells (Figure S3A–C) and the caspase1-specific inhibitor treated BV2 cells, to evaluate proinflammatory cytokine release treated with or without high glucose (35 mM) for 12 h. The results showed that NLRP3 KO decreased the IL-1β proinflammatory cytokine levels, and inhibition of caspase1 also decreased the IL-1β proinflammatory cytokine levels (Figure 3A,C,D). Importantly, neither of these treatments influenced the high glucose-induced expression of TREM2 (Figure 3B). In summary, these findings demonstrated that TREM2 modulates high glucose-induced microglial inflammation via the NLRP3 signaling pathway.

## 4. Discussion

The microglia-mediated inflammatory response is the main pathogenic factor of many neurodegenerative diseases. In diabetes, sustained high glucose stimulation leads to the overactivation of microglia, which exacerbates neuroinflammation. However, the mechanism underlying the activation of microglia exposed to hyperglycemia remains unclear. The present study demonstrated that TREM2 modulates high glucose-induced microglial inflammation via the NLRP3 signaling pathway providing evidence for the study of chronic neuroinflammation and the immunometabolic response in diabetes neuroinflammation.

Previous studies have demonstrated that hyperglycemia activates the innate immune response mediated by TLR2, TLR4, and the NLRP3 inflammasome, inducing the production of various proinflammatory cytokines, including IL-1β, IL18, IL-6, and TNFα [25]. Suppression of inflammation may ameliorate diabetes and diabetes complications [26,27]. In microglia, a high-glucose state may lead to increases in the levels of proinflammatory cytokines, such as TNF-αand IL-6, and decreases in anti-inflammatory cytokines, such as IL-10. High glucose stimulates TNFα and MCP-1 expression in rat microglia via the ROS and NF-κB pathways [15]. High glucose also stimulates GRO secretion from rat microglia via the ROS, PKC, and NF-κB pathways [28]. Others have found that lncRNA MALAT1 promotes the high glucose-induced inflammatory response of microglial cells by provoking MyD88/IRAK1/TRAF6 signaling [29]. In diabetic retinopathy, under high glucose conditions, although the number of microglial cells decreased, they showed a less ramified morphology, suggesting a more activated state, as indicated by upregulation of the levels of microglial activation marker ED-1. The researchers proved that IL-1β plays an important role in retinal microglia activation and proliferation under diabetes [30]. Furthermore, the IL-1β inhibitor, canakinumab, has been reported to reduce incident diabetes [31]. Our study found that high glucose-stimulated the expression of the IL-1β proinflammatory cytokine and TREM2. TREM2 has been reported to play a critical role in microglia-mediated neuroprotective function in the central nervous system. Because the molecular function of TREM2 in regulating microglia-mediated diabetes neuroinflammation remains unclear, elucidating the molecular pathway of TREM2 in hyperglycemia-induced inflammation response is critical for the prevention and treatment of diabetes neuroinflammation. A recent study has found that downregulation of TREM2 expression inhibits the release of inflammatory factors from LPS-stimulated microglia by inhibiting NF-κB signaling pathway activity [32]. TREM2 knockout mice produced lower inflammatory cytokine levels and reduced bacterial killing and T-cell activation than cells from wild-type mice in the inflammatory bowel disease model [33]. TREM2 knockout alveolar macrophages displayed augmented bacterial phagocytosis in vitro and in vivo compared to WT alveolar macrophages [34]. On the other hand, researchers have found that TREM2 suppresses inflammation by attenuating microglial activation [32], suppressing PI3K/NFκB signaling [35,36], downregulating TLR signaling [37,38,39], and modulating the TREM2-autophagy axis [39]. TREM2 also modifies the microglial phenotype and provides neuroprotection in P301S tau transgenic mice [40]. Based on previous studies, TREM2 exerts pro-inflammatory or anti-inflammatory effects depending on the different stimulus and disease context, and the mechanism needs to be further investigated. Because the expression of TREM2 and its regulatory effect on high glucose-induced inflammation are still unclear, our study aimed to elucidate the molecular function of TREM2 in high glucose-induced microglial inflammation. We first demonstrated that high glucose increased the transcription and expression level of TREM2 after treatment for 8 h. Then, using CRISPR/Cas9-mediated TREM2 KO and PCDNA3.1-mediated TREM2 OE BV2 cells, we detected the activation of the IL1β proinflammatory cytokine. TREM2 KO reduced the IL-1β proinflammatory cytokine levels, and TREM2 OE elevated the IL-1β proinflammatory cytokine levels. Our results showed that TREM2 promotes high glucose-induced microglial inflammation.

We next hypothesized that TREM2 regulates high glucose-induced inflammation via the NLRP3 inflammasome pathway. The NLRP3 inflammasome is a member of the NLR family of innate immune cell sensors. They are crucial regulators of cytokine secretions, promoting neuroinflammation and insulin resistance [41]. Evidence has shown that the NLRP3 inflammasome, IL-1β, thioredoxin-interacting protein (TXNIP), and pyroptosis play vital roles in the development of diabetes [42]. Therefore, it is interesting to clarify the relationship between TREM2 and NLRP3. A recent study found that *P. aeruginosa* keratitis was more severe in TREM2-/- versus wild type C57B/6 mice, as indicated by increased clinical scores, bacterial load, and cornea pathology. The exacerbated disease progression caused by TREM2 deficiency was associated with boosted activation of caspase-1 and subsequent pyroptosis and increased expression of IL-1β. The authors also found that TREM2 co-immunoprecipitated with procaspase-1 and NLRP3 in BMDMs treated with HK-PA, LPS, or nigericin, as well as *P. aeruginosa*-infected mouse corneas. The coimmunoprecipitation of NLRP3 and TREM2 suggests that TREM2 may regulate inflammasome activation by direct interaction. In this study, the authors mainly demonstrated that caspase-1 dependent pyroptosis was responsible for more serious tissue pathological injuries and increased bacterial load. They revealed a novel mechanism by which TREM2 mediates the immune defense against *P. aeruginosa* [43]. Our study mainly investigated the high glucose-stimulated microglia inflammation, different from *P. aeruginosa* induced acute inflammatory response. High glucose induces metabolic stress may cause a low-grade chronic inflammatory response. In this process, we found that TREM2 mediates the inflammatory response via interacting with NLRP3. We found that high glucose promoted the interaction of TREM2 and NLRP3, thereby activating the NLRP3 inflammasome. TREM2 KO attenuated the interaction, and TREM2 OE enhanced the interaction. Moreover, TREM2 KO suppressed the activation of the NLRP3 inflammasome, while TREM2 OE augmented NLRP3 inflammasome activation. Our results indicated that high glucose promotes the elevated expression of TREM2, which activates the NLRP3 inflammasome signaling pathway.

NLRP3 inflammasome inhibition has been reported to improve diabetes-mediated cognitive impairment [44]. However, the endogenous regulatory mechanisms remain unclear. Several chemicals have been reported to ameliorate neuroinflammation by inhibiting the NLRP3 pathway. For example, MitoQ inhibits the NLRP3 inflammasome, promoting a shift in microglia toward the M2 phenotype in intracerebral hemorrhage-induced brain damage [45]. Salvianolic acids alleviate cerebral ischemia/reperfusion injury by inhibiting NLRP3 inflammasomes in microglia [46]. Glycyrrhizin also inhibits NLRP3 inflammasome activation and promotes microglia to undergo M2 polarization after traumatic spinal cord injury [47]. Our study found that blocking NLRP3 signaling ameliorated high glucose-induced neuroinflammation, indicating that TREM2 suppresses high glucose-induced neuroinflammation via the NLRP3 signaling pathway.

## 5. Conclusions

The present study found that TREM2 regulates high glucose-induced neuroinflammation via the NLRP3 signaling pathway. This provides evidence for the study of diabetes-induced chronic neuroinflammation.

## Data Availability

Data is contained within the article or supplementary material.

## Supplementary Materials

The following are available online at https://www.mdpi.com/article/10.3390/brainsci11070896/s1, Figure S1: CRISPR/Cas9 mediated knockout of TREM2 in BV2 cells; Figure S2: Construction of TREM2 overexpression BV2 cell line; Figure S3. CRISPR/Cas9 mediated knockout of NLRP3 in BV2 cells.

## Abbreviations

TREM2	triggering receptor expressed on myeloid cells-2;
NLRP3	nucleotide-binding oligomerization domain-like receptor protein 3;
qPCR	real-time quantitative polymerase chain reaction;
ELISA	enzyme-linked immunosorbent assay;
Co-IP	co-Immunoprecipitation;
IL-1β	interlukin-1β;
KO	knockout;
OE	overexpression;
ROS	reactive oxygen species;
DAP12	DNAX activation protein 12;
NHD	Nasu-Hakola disease;
TLR2	TLR4, toll like receptor2,4;
LPS	lipopolysaccharide;
PLOSL	Polycystic lipomembranous osteodysplasia with sclerosing leukoencephalopathy;
NEB	New England Biolabs;

## References

[1] Kahn S.E., Cooper M.E., Del Prato S. (2014). Pathophysiology and treatment of type 2 diabetes: Perspectives on the past, present, and future. Lancet.

[2] Yu Z.-W., Zhang J., Li X., Wang Y., Fu Y.-H., Gao X.-Y. (2020). A new research hot spot: The role of NLRP3 inflammasome activation, a key step in pyroptosis, in diabetes and diabetic complications. Life Sci..

[3] Diedisheim M., Carcarino E., Vandiedonck C., Roussel R., Gautier J.-F., Venteclef N. (2020). Regulation of inflammation in diabetes: From genetics to epigenomics evidence. Mol. Metab..

[4] Bing C. (2015). Is interleukin-1beta a culprit in macrophage-adipocyte crosstalk in obesity?. Adipocyte.

[5] Zhang Y., Feng S., Nie K., Li Y., Gao Y., Gan R. (2018). TREM2 modulates microglia phenotypes in the neuroinflammation of Parkinson’s disease. Biochem. Biophys. Res. Commun..

[6] Tang Y., Le W. (2016). Differential Roles of M1 and M2 Microglia in Neurodegenerative Diseases. Mol. Neurobiol..

[7] Xu Q., Xu W., Cheng H., Yuan H., Tan X. (2019). Efficacy and mechanism of cGAMP to suppress Alzheimer’s disease by elevating TREM2. Brain Behav. Immun..

[8] Zhai Q., Li F., Chen X., Jia J., Sun S., Zhou D., Ma L., Jiang T., Bai F., Xiong L. (2017). Triggering Receptor Expressed on Myeloid Cells 2, a Novel Regulator of Immunocyte Phenotypes, Confers Neuroprotection by Relieving Neuroinflammation. Anesthesiology.

[9] Zhang J., Zheng Y., Luo Y., Du Y., Zhang X., Fu J. (2019). Curcumin inhibits LPS-induced neuroinflammation by promoting microglial M2 polarization via TREM2/TLR4/NF-kappaB pathways in BV2 cells. Mol. Immunol..

[10] He G.-L., Luo Z., Shen T.-T., Wang Z.-Z., Li P., Luo X., Yang J., Tan Y.-L., Wang Y., Gao P. (2020). TREM2 Regulates Heat Acclimation-Induced Microglial M2 Polarization Involving the PI3K-Akt Pathway Following EMF Exposure. Front. Cell. Neurosci..

[11] Minami Y., Sonoda N., Hayashida E., Makimura H., Ide M., Ikeda N. (2018). p66Shc Signaling Mediates Diabetes-Related Cogni-tive Decline. Sci. Rep..

[12] Klein J.P., Hains B.C., Craner M.J., Black J.A., Waxman S.G. (2004). Apoptosis of vasopressinergic hypothalamic neurons in chronic dia-betes mellitus. Neurobiol. Dis..

[13] Musen G., Lyoo I.K., Sparks C.R., Weinger K., Hwang J., Ryan C.M., Jimerson D.C., Hennen J., Renshaw P.F., Jacobson A.M. (2006). Effects of Type 1 Diabetes on Gray Matter Density as Measured by Voxel-Based Morphometry. Diabetes.

[14] Wang J.-Y., Yang J.-M., Wang J.-Y., Tao P.-L., Yang S.N. (2001). Synergistic apoptosis induced by bacterial endotoxin lipopolysaccharide and high glucose in rat microglia. Neurosci. Lett..

[15] Quan Y., Jiang C.T., Xue B., Zhu S.G., Wang X. (2011). High glucose stimulates TNFalpha and MCP-1 expression in rat microglia via ROS and NF-kappaB pathways. Acta Pharmacol. Sin..

[16] Krady J.K., Basu A., Allen C.M., Xu Y., LaNoue K.F., Gardner T.W., Levison S. (2005). Minocycline Reduces Proinflammatory Cytokine Expression, Microglial Activation, and Caspase-3 Activation in a Rodent Model of Diabetic Retinopathy. Diabetes.

[17] Qin Q., Teng Z., Liu C., Li Q., Yin Y., Tang Y. (2021). TREM2, microglia, and Alzheimer’s disease. Mech. Ageing Dev..

[18] Yeh F.L., Hansen D.V., Sheng M. (2017). TREM2, Microglia, and Neurodegenerative Diseases. Trends Mol. Med..

[19] Filipello F., Morini R., Corradini I., Zerbi V., Canzi A., Michalski B. (2018). The Microglial Innate Immune Receptor TREM2 Is Re-quired for Synapse Elimination and Normal Brain Connectivity. Immunity.

[20] Jaitin D.A., Adlung L., Thaiss C.A., Weiner A., Li B., Descamps H. (2019). Lipid-Associated Macrophages Control Metabolic Ho-meostasis in a Trem2-Dependent Manner. Cell.

[21] Ulland T.K., Song W.M., Huang S.C., Ulrich J.D., Sergushichev A., Beatty W.L. (2017). TREM2 Maintains Microglial Metabolic Fit-ness in Alzheimer’s Disease. Cell.

[22] Gritsenko A., Green J.P., Brough D., Lopez-Castejon G. (2020). Mechanisms of NLRP3 priming in inflammaging and age related dis-eases. Cytokine Growth Factor Rev..

[23] Li Y., Wang X., Xu H., Wang C., An Y., Luan W., Wang X., Li S., Ma F., Ni L. (2018). Cordycepin Modulates Body Weight by Reducing Prolactin Via an Adenosine A1 Receptor. Curr. Pharm. Des..

[24] Ran F.A., Hsu P.D., Wright J., Agarwala V., Scott D.A., Zhang F. (2013). Genome engineering using the CRISPR-Cas9 system. Nat. Protoc..

[25] Wada J., Makino H. (2016). Innate immunity in diabetes and diabetic nephropathy. Nat. Rev. Nephrol..

[26] Karstoft K., Pedersen B.K. (2015). Exercise and type 2 diabetes: Focus on metabolism and inflammation. Immunol. Cell Biol..

[27] Verma S., Mathew V., Farkouh M.E. (2018). Targeting Inflammation in the Prevention and Treatment of Type 2 Diabetes: Insights From CANTOS. J. Am. Coll. Cardiol..

[28] Quan Y., Du J., Wang X. (2007). High glucose stimulates GRO secretion from rat microglia via ROS, PKC, and NF-κB pathways. J. Neurosci. Res..

[29] Wang L.Q., Zhou H.J. (2018). LncRNA MALAT1 promotes high glucose-induced inflammatory response of microglial cells via pro-voking MyD88/IRAK1/TRAF6 signaling. Sci. Rep..

[30] Baptista F.I., Aveleira C.A., Castilho A.F., Ambrosio A.F. (2017). Elevated Glucose and Interleukin-1beta Differentially Affect Retinal Microglial Cell Proliferation. Mediat. Inflamm..

[31] Everett B.M., Donath M.Y., Pradhan A.D., Thuren T., Pais P., Nicolau J., Glynn R.J., Libby P., Ridker P.M. (2018). Anti-Inflammatory Therapy With Canakinumab for the Prevention and Management of Diabetes. J. Am. Coll. Cardiol..

[32] Wang M., Gao X., Zhao K., Chen H., Xu M., Wang K. (2019). Effect of TREM2 on Release of Inflammatory Factor from LPS-stimulated Microglia and Its Possible Mechanism. Ann. Clin. Lab. Sci..

[33] Correale C., Genua M., Vetrano S., Mazzini E., Martinoli C., Spinelli A., Arena V., Peyrin-Biroulet L., Caprioli F., Passini N. (2013). Bacterial Sensor Triggering Receptor Expressed on Myeloid Cells-2 Regulates the Mucosal Inflammatory Response. Gastroenterology.

[34] Sharif O., Gawish R., Warszawska J.M., Martins R., Lakovits K., Hladik A. (2014). The triggering receptor expressed on myeloid cells 2 inhibits complement component 1q effector mechanisms and exerts detrimental effects during pneumococcal pneumo-nia. PLoS Pathog..

[35] Li C., Zhao B., Lin C., Gong Z., An X. (2019). TREM2 inhibits inflammatory responses in mouse microglia by suppressing the PI3K/NF-kappaB signaling. Cell Biol. Int..

[36] Zhu Z., Zhang X., Dong W., Wang X., He S., Zhang H. (2020). TREM2 suppresses the proinflammatory response to facilitate PRRSV infection via PI3K/NF-kappaB signaling. PLoS Pathog..

[37] Long H., Zhong G., Wang C., Zhang J., Zhang Y., Luo J. (2019). TREM2 Attenuates Abeta1-42-Mediated Neuroinflammation in BV-2 Cells by Downregulating TLR Signaling. Neurochem. Res..

[38] Zhou J., Yu W., Zhang M., Tian X., Li Y., Lu Y. (2019). Imbalance of Microglial TLR4/TREM2 in LPS-Treated APP/PS1 Transgenic Mice: A Potential Link between Alzheimer’s Disease and Systemic Inflammation. Neurochem. Res..

[39] Wang Y., Shi Y., Huang Y., Liu W., Cai G., Huang S. (2020). Resveratrol mediates mechanical allodynia through modulating in-flammatory response via the TREM2-autophagy axis in SNI rat model. J. Neuroinflammation..

[40] Jiang T., Zhang Y.D., Chen Q., Gao Q., Zhu X.C., Zhou J.S. (2016). TREM2 modifies microglial phenotype and provides neuropro-tection in P301S tau transgenic mice. Neuropharmacology.

[41] Wan Z., Fan Y., Liu X., Xue J., Han Z., Zhu C., Wang X. (2019). NLRP3 inflammasome promotes diabetes-induced endothelial inflammation and atherosclerosis. Diabetes Metab. Syndr. Obes. Targets Ther..

[42] Ding S., Xu S., Ma Y., Liu G., Jang H., Fang J. (2019). Modulatory Mechanisms of the NLRP3 Inflammasomes in Diabetes. Biomol..

[43] Qu W., Wang Y., Wu Y., Liu Y., Chen K., Liu X. (2018). Triggering Receptors Expressed on Myeloid Cells 2 Promotes Corneal Re-sistance Against Pseudomonas aeruginosa by Inhibiting Caspase-1-Dependent Pyroptosis. Front. Immunol..

[44] Ward R., Li W., Abdul Y., Jackson L., Dong G., Jamil S. (2019). NLRP3 inflammasome inhibition with MCC950 improves diabe-tes-mediated cognitive impairment and vasoneuronal remodeling after ischemia. Pharmacol. Res..

[45] Chen W., Guo C., Huang S., Jia Z., Wang J., Zhong J., Ge H., Yuan J., Chen T., Liu X. (2020). MitoQ attenuates brain damage by polarizing microglia towards the M2 phenotype through inhibition of the NLRP3 inflammasome after ICH. Pharmacol. Res..

[46] Ma D.C., Zhang N.N., Zhang Y.N., Chen H.S. (2021). Salvianolic Acids for Injection alleviates cerebral ischemia/reperfusion injury by switching M1/M2 phenotypes and inhibiting NLRP3 inflammasome/pyroptosis axis in microglia in vivo and in vitro. J. Eth-nopharmacol..

[47] Su X.-Q., Wang X.-Y., Gong F.-T., Feng M., Bai J.-J., Zhang R.-R., Dang X.-Q. (2020). Oral treatment with glycyrrhizin inhibits NLRP3 inflammasome activation and promotes microglial M2 polarization after traumatic spinal cord injury. Brain Res. Bull..

