# Supplementary material for: TREM2 Regulates High Glucose-Induced Microglial Inflammation via the NLRP3 Signaling Pathway
Source: Brain Sci. 2021 Jul 7;11(7):896. doi: 10.3390/brainsci11070896 (PMC8306970; doi:10.3390/brainsci11070896)
Supplement: Supplementary file 1 [file brainsci-11-00896-s001.zip › Supplemeantary figure legends.pdf]

Supplementary Figure legends

Fig S1. CRISPR/Cas9 mediated knockout of TREM2 in BV2 cells. (A) Schematic diagram of the px459 plasmid used for TREM2 and NLRP3 gene knockout with CRISPR/Cas9 technology. (B) Schematic diagram of sgRNA targeting murine TREM2. The target sites of the sgRNA are highlighted in red, and the protospacer-adjacent motif (PAM) sequence is highlighted in blue. (C, D) Mutation detection in TREM2-KO cells by western blotting. TREM2 expression was significantly decreased in KO cells compared with WT control cells (*** $p< 0.001$).

Fig S2. Construction of TREM2 overexpression BV2 cell line. (A) Schematic diagram of the overexpression plasmid pCDNA3.1+3*flag. (B) Schematic diagram of the TREM2 overexpression plasmid pCDNA3.1+TREM2 3*flag. (C, D) Verification of TREM2 OE by western blotting; the results were normalized to GAPDH (*** $p< 0.001$).

Fig S3. CRISPR/Cas9 mediated knockout of NLRP3 in BV2 cells (A) Schematic diagram of sgRNA targeting murine NLRP3. The target sites of the sgRNA are highlighted in red, and the protospacer-adjacent motif (PAM) sequence is highlighted in blue. (B, C) Mutation detection in NLRP3-KO cells by western blotting. NLRP3 expression was significantly decreased in KO cells compared with WT control cells (***$p< 0.001$).
