# Supplementary material for: TREM2 Regulates High Glucose-Induced Microglial Inflammation via the NLRP3 Signaling Pathway
Source: Brain Sci. 2021 Jul 7;11(7):896. doi: 10.3390/brainsci11070896 (PMC8306970; doi:10.3390/brainsci11070896)
Supplement: Supplementary file 1 [file brainsci-11-00896-s001.zip › Supplementary materials.pptx]

## Slide 1
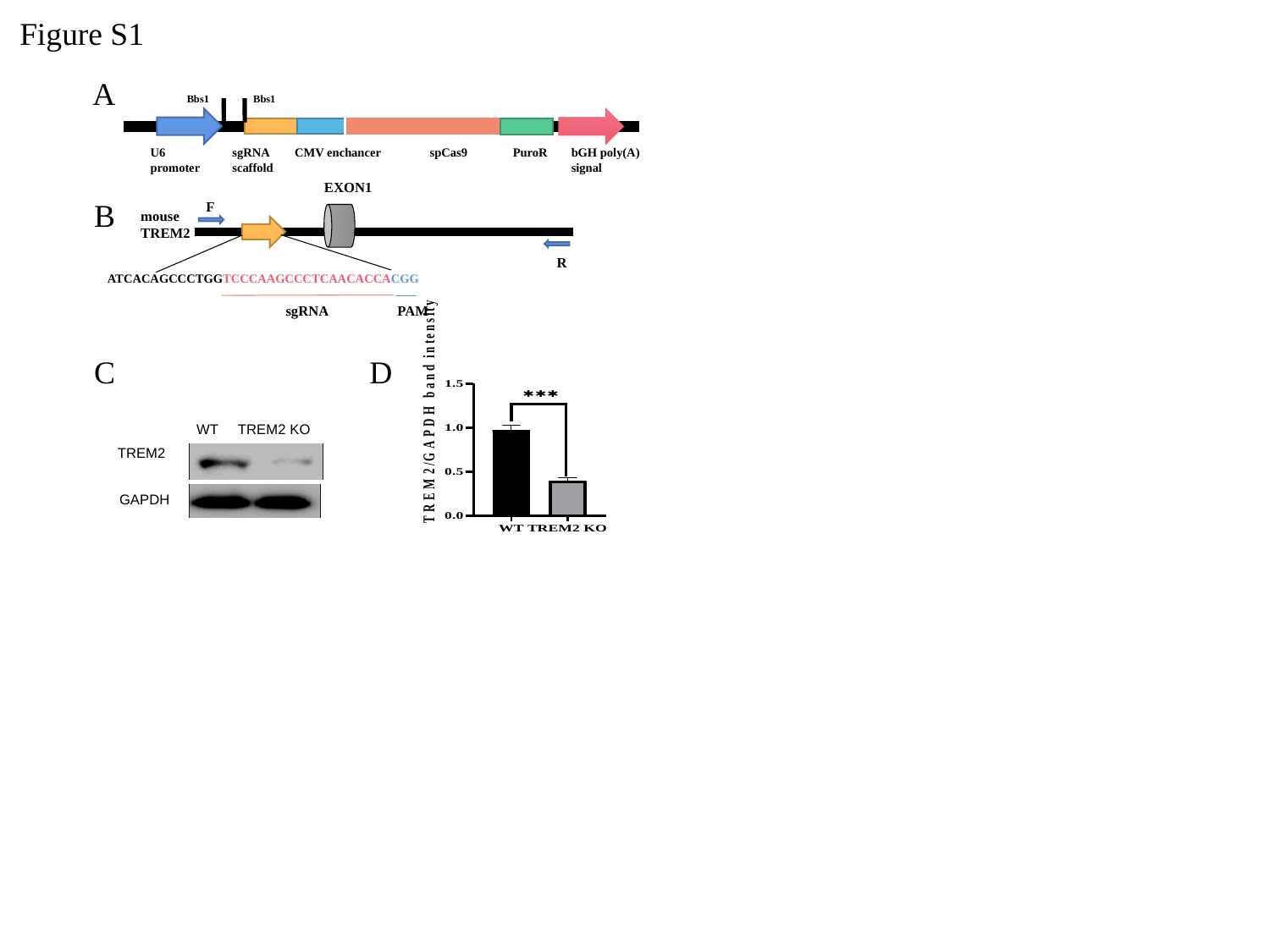

Figure S1
A
Bbs1
Bbs1
U6 promoter
sgRNA scaffold
CMV enchancer
spCas9
PuroR
bGH poly(A) signal
EXON1
F
mouse TREM2
R
 ATCACAGCCCTGGTCCCAAGCCCTCAACACCACGG
sgRNA
PAM
B
C
D
 WT TREM2 KO
TREM2
GAPDH

## Slide 2
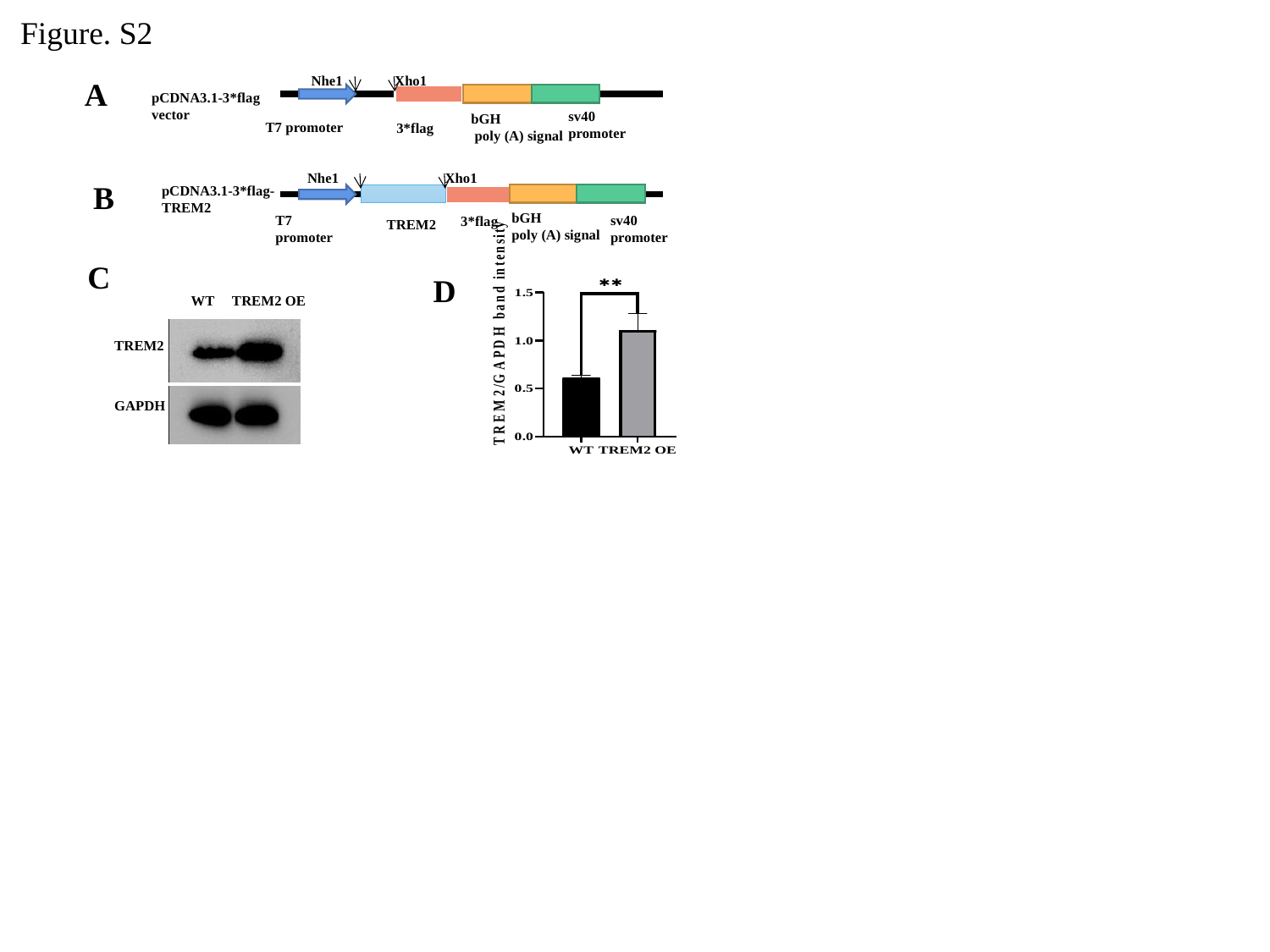

Figure. S2
Nhe1
Xho1
pCDNA3.1-3*flag
vector
sv40
promoter
bGH
 poly (A) signal
T7 promoter
3*flag
Nhe1
Xho1
pCDNA3.1-3*flag-TREM2
bGH
poly (A) signal
T7 promoter
sv40 promoter
3*flag
TREM2
A
B
C
D
 WT TREM2 OE
TREM2
GAPDH

## Slide 3
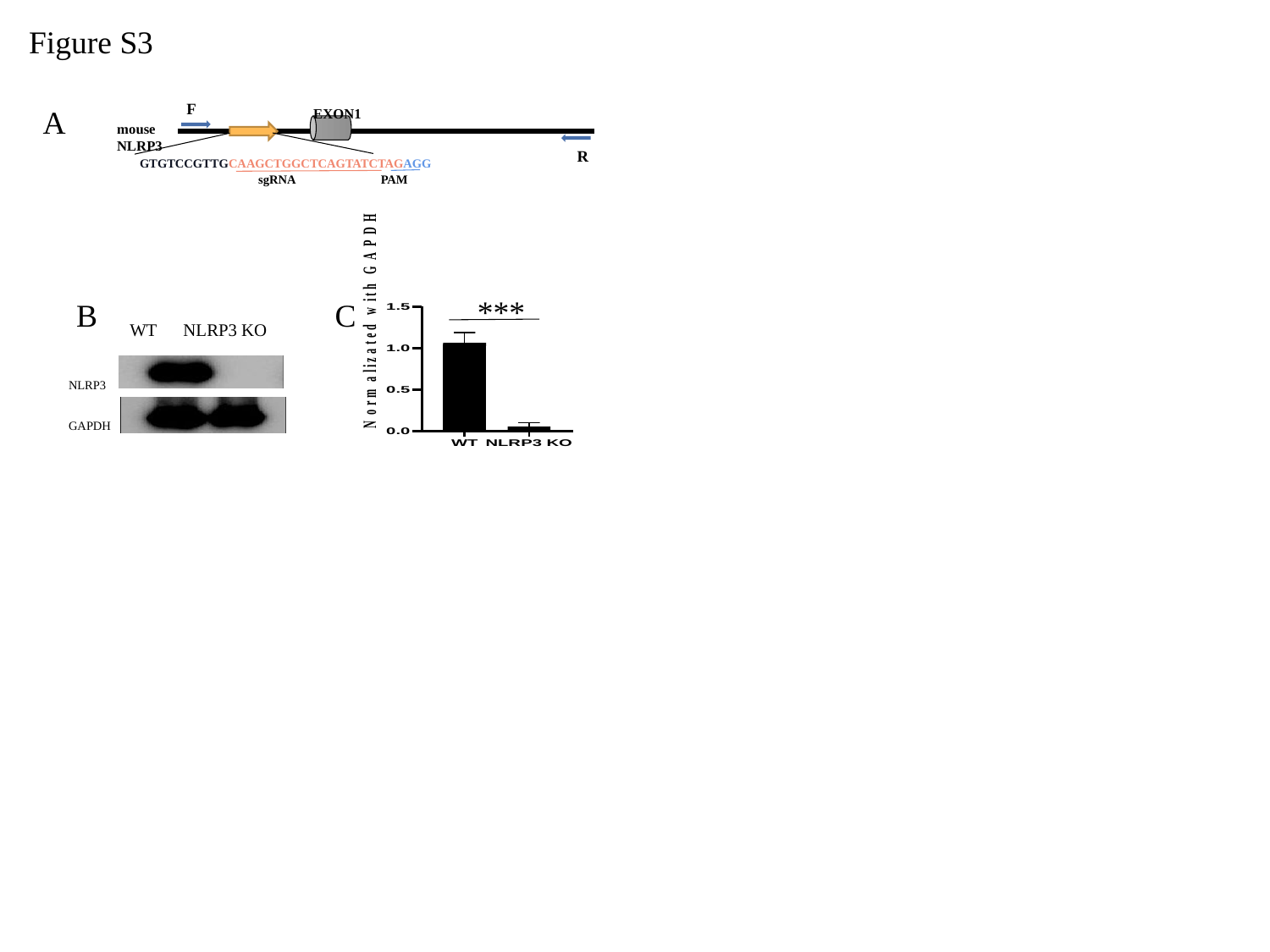

Figure S3
F
EXON1
mouse NLRP3
R
GTGTCCGTTGCAAGCTGGCTCAGTATCTAGAGG
sgRNA
PAM
A
***
B
C
 WT NLRP3 KO
NLRP3
GAPDH
